# A Study of the Friction Stir Lap Welding of AA5052 and Polypropylene

**DOI:** 10.3390/polym15234481

**Published:** 2023-11-21

**Authors:** Ahmed I. Alhatti, Jamal Sheikh-Ahmad, Fahad Almaskari, Kamran A. Khan, Suleyman Deveci, Abdelrahman I. Hosny

**Affiliations:** 1 Department of Mechanical Engineering, Khalifa University of Science and Technology, Abu Dhabi P.O. Box 127788, United Arab Emirates; 100059802@ku.ac.ae (A.I.A.); jamal.sheikh-ahmad@wne.edu (J.S.-A.); 100060586@ku.ac.ae (A.I.H.); 2 Department of Mechanical Engineering, Western New England University, Springfield, MA 01119, USA; 3 Department of Aerospace Engineering, Khalifa University of Science and Technology, Abu Dhabi P.O. Box 127788, United Arab Emirates; kamran.khan@ku.ac.ae; 4 Borouge Pte. Ltd., Abu Dhabi P.O. Box 6951, United Arab Emirates

**Keywords:** friction stir lap welding, dissimilar materials welding, polypropylene, AA5052, metal-polymer lap joint, lap tensile strength, tool design

## Abstract

Friction stir lap welding (FSLW) remains a pioneering technique for creating hybrid joints between AA5052 aluminium alloy and polypropylene (PP), particularly with the metal-on-top configuration. Building upon previous research, this study introduces a tapered fluted pin tool design and investigates its effectiveness in the welding process. Our results, supported by ANOVA, chemical, and microstructural analyses, reiterate that the optimal welding parameters stand at a rotational speed of 1400 RPM and a traverse speed of 20 mm/min. This combination produces a joint tensile strength of 3.8 MPa, signifying 16.54% of the weaker material’s inherent strength. Microstructural evaluations revealed a unique composite of aluminium chips intermeshed with PP, strengthened further by aluminium hooks. Crucially, mechanical interlocking plays a predominant role over chemical bonding in achieving this joint strength. The study underscores the absence of significant C-O-Al bonds, hinting at the PP degradation without the thermo-oxidation process. Additionally, joint strength was found to inversely correlate with the interaction layer’s thickness. The findings fortify the promise of FSLW with the novel fluted pin design for enhancing joints between AA5052 and PP, emphasising the potential of mechanical interlocking as a principal factor in achieving high-quality welds.

## 1. Introduction

In the future, space tourism will become more accessible to the public. In parallel, the manufacturing industry seeks innovative approaches to enhance mechanical components in sectors like robotics and automation, driving a need for solutions such as complex hybrid structures made of metal and polymers. Polymers offer high specific strength, fatigue resistance, fracture strength, and low thermal expansion [1]. They are lightweight and highly formable, making them suitable for use in the aerospace and automotive industries [2]. Additionally, aluminium alloys are widely employed in automotive and aerospace applications due to their favourable strength-to-weight ratio, cost-effectiveness, corrosion resistance, and low density [3,4].

Friction stir welding of metal and polymer is made possible because of mechanical interlocking and chemical bonding [5,6]. The formation of metallic arrows in the interface of the joint is related to the flow of the material [7]. Material flow is controlled by the pin shape. Friction stir welding (FSW) is a novel method for joining dissimilar materials. This process primarily relies on a non-consumable rotating tool, which exerts a thermo-mechanical effect on the specimen as it presses down and traverses through the materials [8]. There are other processes derived from FSW. As the tool presses on the specimen, it generates heat through friction and plastic deformation. The plasticised soft materials then mix, forge, and interlock, resulting in a unique weld nugget. Friction stir welding can be divided into three stages: tool plunge, dwell period, and welding. The tool plunge stage involves pressing the tool pin into the joint section of the specimens. After this, the tool rotates without traversing, maintaining contact with the workpiece to generate heat. This process, which plasticizes the materials before movement, is known as the dwell period. The final stage, welding, sees the tool traverse along the joint line, where the actual welding of the materials takes place [9].

Friction stir welding of metal and polymer is feasible due to mechanical interlocking and chemical bonding [5,6]. The presence of metallic arrows at the joint interface is linked to material flow [7]. Furthermore, this material flow is governed by the pin’s shape. When a thread is used on the pin, the aluminium moves downward, depending on the pin’s rotational direction. The soft polymer facilitates penetration at the intersection of the specimens [10,11]. The aluminium anchor is located in the thermo-mechanically affected zone (TMAZ). These anchors are commonly referred to as aluminium arrows [6]. The distribution of the Al arrows differs between the advancing side and the retreating side. On the advancing side, the anchor is noticeably larger than on the retreating side. This is due to the heat input on the advancing side adequately softening the aluminium, leading to a larger anchor [10]. The size of the anchor is influenced by the traverse speed of the tool. Faster movement tends to produce coarser aluminium fragments [12]. As the size of the Al anchor increases, there is a positive change in the tensile property of the weld. Various material combinations have been tested in the past, leading to a range of welding qualities. When using lap welding with PC on top, 68% of the material’s shear strength was achieved [13], while placing Al on top resulted in 21% of the material’s shear strength [10].

Heat input is directly related to the shear strength of the weld. Understanding the heat generated during welding provides a clearer insight into the joining process. Factors such as tool pin geometry, the interface between surfaces, and the thermal conductivity of the specimens influence heat generation in various ways [14]. Moreover, the tool rotation speed, a crucial process parameter, exerts the most significant impact on heat generation [11]. Traverse speed is another vital process parameter. Decreasing it prolongs the tool’s contact time with the specimen, leading to increased heat generation [15]. Excessive heat input can amplify the hook defect, subsequently diminishing joint strength [16]. This phenomenon transpires beyond a specific heat input threshold. Due to peak temperature and the coefficient of thermal expansion, a layer primarily composed of O, C, and Al forms at the interface. The adhesive force between this layer and the interface is weak, resulting in a gap. As heat input escalates, both the layer’s thickness and the gap between it and the two interfaces increase. Consequently, an increase in heat input correlates with reduced shear strength [16].

The quality of the weld is influenced by both the tool design and the process parameters. Different parameters have distinct effects on the weld configuration. In lap welding with metal on top, specific relationships arise depending on the tool used. Shahmiri et al. [16] confirmed that increasing the rotation speed led to a thicker reaction layer. This phenomenon is due to the altered heat input when the rotation speed was adjusted from 800 to 1200 RPM. Consequently, a thicker reaction layer reduces the shear–tensile load of the joint.

Dalwadi et al. [17] experimented with varying rotation speeds, ranging from 500 to 1000 rpm, and traverse speeds between 31.5 to 50 mm/min. Their findings revealed that as the rotation speed increased, the joint strength decreased when the traverse speed was below 40 mm/min. However, when the traverse speed reached 40 mm/min, the joint strength began to increase with a rising rotation speed. Beyond this traverse speed, the joint strength started to decline with further increases in rotation speed. Additionally, Dalwadi et al. [17] observed that elevating the traverse speed generally enhanced joint strength across all rotation speeds. Yet, once a particular traverse speed was surpassed, the joint strength began to decline.

Huang et al. [10] proposed that the strength of a joint is directly tied to the presence of an aluminium anchor embedded within the polymer. This “interlock effect” elucidates the relationship between traverse speed and strength. Increasing the traverse speed continuously results in reduced preheating of the joint’s temperature and a shortened duration of material contact with the tool. This scenario leads to suboptimal material mixing, ultimately causing the aluminium anchor to contract. When the traverse speed was increased from 30 mm/min to 90 mm/min, there was an initial enhancement in bond strength. However, after surpassing a specific speed, the strength began to wane with additional increases in traverse speed.

The tool primarily comprises two components: the shoulder and the pin. Minor adjustments to the pin and shoulder profiles can significantly impact the weld quality [11]. The tool has been designed to facilitate mechanical interlocking by directing the flow of aluminium based on the pin profile. Given that mechanical interlocking is pivotal to weld formation, refining the tool design to optimise this interlocking is essential for superior weld quality [18]. The stirring pin can produce both frictional and deformational heat. Additionally, it disrupts and shears the workpiece material, aiding in its mixing behind the tool [19]. The pin boasts two specific geometries: its overall shape and its end face. Various shapes, including cylindrical, triangular, rectangular, and tapered, are available. In some contexts, additional features like threads or grooves can be incorporated into the pin to enhance mixing and control material flow more effectively. For example, a left-hand thread combined with a clockwise rotation pushes the material downward, driven by the thread’s rotation. This design ensures that the material circles the pin several times before settling behind it, promoting void closure, material stirring, and oxide breakdown [19]. A tapered pin without threading has proven counterproductive for joint formation. In contrast, a threaded tapered pin consistently yields high-quality welds with optimal joint integrity [20]. To further improve mixing, modifications can be made to the pin profile, increasing the contact area [21].

Shahmiri et al. [16] conducted experiments using two tools with pin lengths of 3.5 mm and 2.5 mm, respectively. The shoulder had a diameter of 20 mm, and the pin was both threaded and tapered, ranging from 4.5 mm at its widest to 2.5 mm. Their aim was to weld AA-5052-H34 with PP-C30S. They achieved a shear strength of 5.1 MPa, roughly 20% of PP-C30S’s inherent shear strength. Notably, the tool with the shorter pin produced joints of significantly lower strength due to insufficient mixing of the Al and polymer, to the extent that these joints could easily be separated by hand. In another study, Upadhyay et al. [22] assessed the implications of adding a scribe to the pin tip during welding, maintaining all other variables. They varied scribe heights between 0.71 and 0.91 mm. Their research indicated that the resultant joint strength was about half that of the weaker material involved. Tests were executed in a lap configuration, positioning the polymer atop AA5182.

The current state of FSLW has shown commendable joint quality for a range of dissimilar metal-to-polymer combinations. Optimal results have been observed when the polymer is positioned on top, because placing the alloy on top presents challenges in metal penetration. Consequently, the opportunities for chemical bonding are restricted in these joints. Moreover, there has been a limited amount of research on the influence of fluted pin tools, like the A-skew pin and triflute pin, especially when discussing FSLW for dissimilar metal–polymer joints. Given the lack of thorough investigations, especially concerning the pairing of polypropylene and AA5052 alloy, which is still in need of enhancement in joint quality through FSLW, there is a compelling reason to delve into the usage of fluted pin tool designs in these processes.

Furthermore, using a lap configuration with aluminium situated above the polymer can potentially lead to improved joint properties due to the direct contact with the shoulder. Thus, pairing aluminium alloy and polypropylene in this manner is promising, especially considering the higher melting point of aluminium alloy compared to polypropylene. This difference in melting points is likely to offer greater control during the welding process.

This study aims to comprehend the core mechanisms involved in joining the AA5052 alloy and polypropylene via friction stir lap welding (FSLW) using a tapered and fluted pin tool. We investigate the effects of changing process parameters, particularly traverse and rotational speed, on aspects such as interface temperature, joint strength, and joint morphology. A comprehensive analysis is conducted using a full factorial design to evaluate the data. Additionally, a correlation between tensile strength, temperatures, and joint morphology is established. The microstructure of the joints is analysed using scanning electron microscopy (SEM) and Raman microscopy to assess both the mechanical and chemical aspects of the joint morphology.

## 2. Materials and Methods

**Figure 1:**
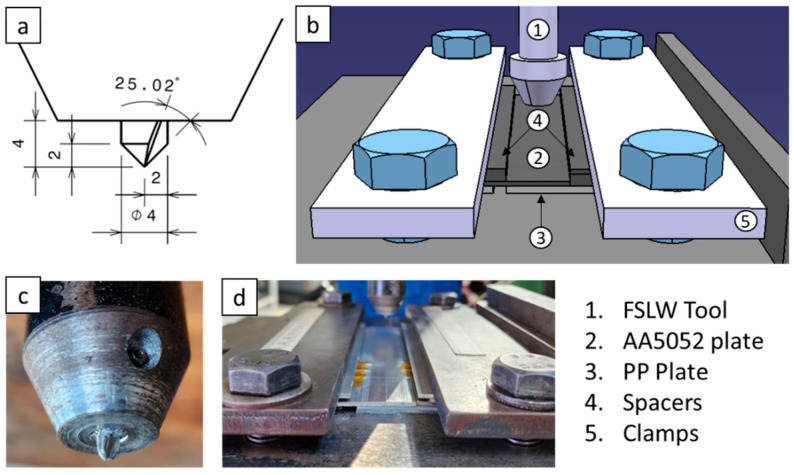
FSW experimental setup. (**a**) FSLW tool, (**b**) setup schematic, (**c**) actual tool, and (**d**) actual setup.

In this study, the materials utilised were a 2 mm-thick aluminium alloy plate on top and a 3 mm-thick polypropylene (PP) plate at the bottom. The physical properties of the AA5052 alloy and polypropylene are presented in Table 1 and Table 2, respectively. Polypropylene (PP) sheets, measuring 330 × 330 × 3 mm, were produced using compression moulding PP pellets with a frame mould. The pellets were initially heated from 25 °C to 210 °C at a rate of 15 °C/min under contact pressure. They were then maintained at 210 °C for 10 min, with the final 5 min under a pressure of 5 MPa. Following this, the samples were cooled to 25 °C at a rate of 15 °C/min under constant pressure. The experimental setup, including the welding tool, is depicted in Figure 1. This setup comprises a Bridgeport milling machine employed for lap joint tests, and an appropriate fixturing attachment for securing the two dissimilar materials. The joint tool design underwent multiple stages of refinement. The ultimate design incorporated a self-tapping screw as a pin; this was divided into two adjustable sections. This design was effectively tested and further optimised and evaluated during the study. The pin used had a diameter and length of 4 mm each and was complemented by a 15 mm rotating shoulder. The effect of the tapered fluted pin on weld quality was assessed by modifying both the rotational and traverse speeds. Mechanical tests were performed to gauge the joint’s strength. Additionally, various characterisation techniques, such as TGA, DSC, RAMAN, EDS, SEM, and optical microscopy, were utilised. The cross-section of the specimen, mechanical hooks, fracture cause, and weld nugget were all closely inspected.

**Figure 2:**
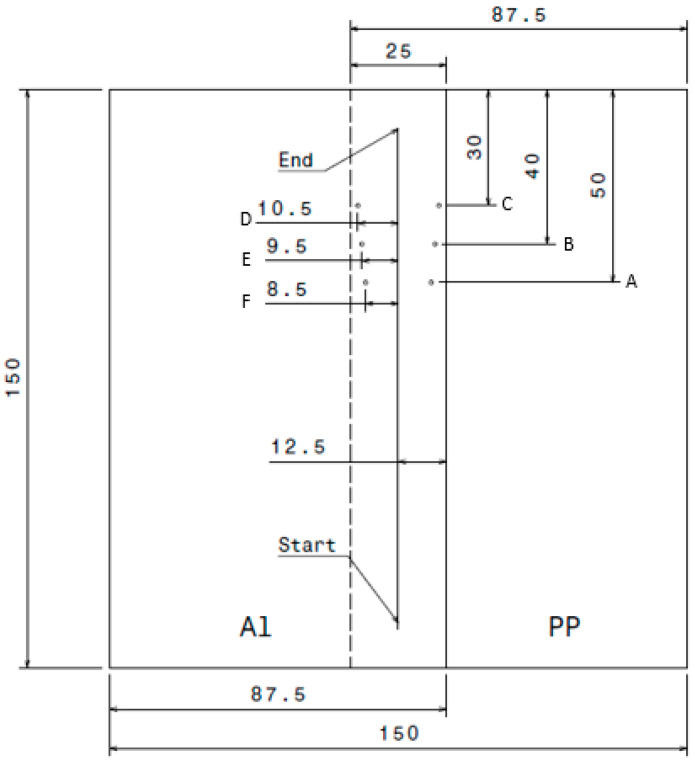
Materials and thermocouples arrangement.

During the friction stir welding (FSW) process, the temperature of the top surface was measured using an IR camera (FLUKE Ti400, Everett, WA, USA). The IR camera’s emissivity was set to 0.59 after calibration. In addition, temperatures at the interface of the two materials were continuously monitored using a K-type Omega 5TC-TT-K-36-72 (OMEGA, Norwalk, CT, USA) precision fine wire, which boasts an accuracy of ±1.5 °C or ±0.25% of the reading. To accommodate these thermocouples, six holes with a diameter of 1 mm were drilled into the aluminium alloy. These holes were strategically positioned along the weld line in 1 mm increments on both sides, as illustrated in Figure 2.

Lap welds were achieved using a full factorial design with two factors, each with three levels, as shown in Table 3. These factors were rotational speed (w) and traverse speed (v). The selection of welding parameters stemmed from preliminary screening experiments conducted prior to this study. Trials ranged from 600 to 1600 rpm and from 10 to 140 mm/min to adapt the tool design and ensure equipment controllability. The ranges selected are based on the highest strengths obtained from tensile testing. The preliminary optical microscopy for those ranges also revealed the most logical joint formations that provide the highest strength, as some testing conditions showed a complete absence of aluminium hooks.

Throughout the experiments, certain parameters remained constant: a clockwise tool rotation, a plunge depth of 0.1 mm, and a tilt angle of 2 degrees. The pin length was consistently maintained at 4 mm from the shoulder, leading to an approximate shoulder penetration of 0.1 mm into the material, depending on the Al plate’s local thickness.

The experiment’s full factorial design consisted of 45 runs, incorporating various combinations of process parameters. This complete design was executed with five replicates. The resulting raw response data were analysed using the Minitab statistical analysis software (Minitab 21 version). Analysis of variance (ANOVA) was conducted at a 95% confidence level, and the significance threshold was established at a probability value (*p*-value) of less than 0.05. This indicates a statistically significant difference between the compared groups. The findings were illustrated using main-effects plots for each response of interest. These responses encompassed maximum load, average load, peak advancing side temperature, peak retreating side temperature, and the highest temperature reading captured by the IR camera.

**Figure 3:**
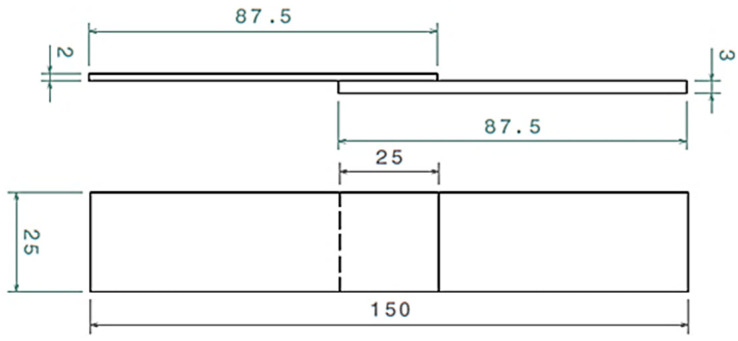
Schematic of shear tensile sample according to ASTM D1002 Standard.

After welding, three rectangular pieces were cut perpendicular to the weld lines. Of these, two specimens were selected for tensile testing. Each tensile specimen had a total length of 150 mm, an overlap of 25 mm, and a width of 25 mm, as shown in Figure 3. The tensile tests were conducted using the Instron 5969 tensile testing machine, which was equipped with a 50 kN load cell. These tests were performed at a crosshead speed of 1 mm/min in accordance with ASTM D1002 standards [23]. Additionally, cross-sectional photographs of each side of the tensile specimen were taken to help identify welding defects such as voids.

To investigate the joint properties, we used Scanning Electron Microscopy (SEM) with the Jeol 7610F machine ( JEOL, Tokyo, Japan). This technique was essential to the study of surface features, interaction layer thickness, hook shapes, voids, and the quality of bonding at the interface. For better imaging of the non-conductive polypropylene (PP) samples, we coated them with gold. These samples were then polished and ground to reveal the weld zone’s microstructure. SEM images were taken at different magnifications to closely examine the weld nugget and its surrounding areas.

Alongside SEM, Optical Microscopy was used to observe the larger structure of the weld. After polishing, the samples were ground again to highlight grain boundaries and check for any defects. The images from this method helped us understand the overall quality of the weld.

We utilised Energy-Dispersive X-ray Spectroscopy (EDS) with the Jeol 7610F machine to determine the elemental composition of the weld zone. With EDS mapping at an acceleration voltage of 5 kV and a working distance of 15 mm, we could see where different elements were in the weld nugget and the heat-affected zone. To investigate the molecular structure and chemical bonds of the polypropylene before and after welding, we used Raman spectroscopy (Horiba, Kyoto, Japan). This was performed using a Horiba Raman microscope that has a 633 nm laser. The resulting Raman spectra were corrected using Origin software (Origin 2023 (10.0) version).

We performed Thermogravimetric Analysis (TGA) using a Q500 TGA machine (TA instruments, New Castle, DE, USA) to test the polypropylene’s thermal stability. The sample was heated at a rate of 10 °C/min from room temperature to 1000 °C in a 99.99% pure nitrogen atmosphere, as per ISO 11358 standards [24]. The Oxidation Induction Time (OIT) was measured using a DSC Q2000 machine (TA instruments, USA). This test started with a 99.99% pure nitrogen gas flow of 50 mL/min. Once the test temperature was reached, the gas was switched to 99.95% pure oxygen at the same flow rate, following ISO 11357-6 [25]. This helped us identify the starting temperature for degradation and measure weight changes at different temperatures.

## 3. Results and Discussion

### 3.1. Tensile Behaviour of the Welded Joint

To determine the variable with the most significant impact on joint strength and quality, experiments were conducted using two specimens for each welded sample, resulting in a total of 90 tensile specimens. The joint quality and strength were analysed based on the average and maximum loads of both specimens using Minitab software (Minitab 21 version). The average load emerged as the primary factor of interest in this study, serving as an indicator of both joint quality and strength.

The results showed a low standard deviation of 15.98, indicating that the data are closely clustered around the regression line. This is further corroborated by the high R-squared value of 91.06%, which suggests that the regression model accounts for a substantial proportion of the variation in the data.

**Figure 4:**
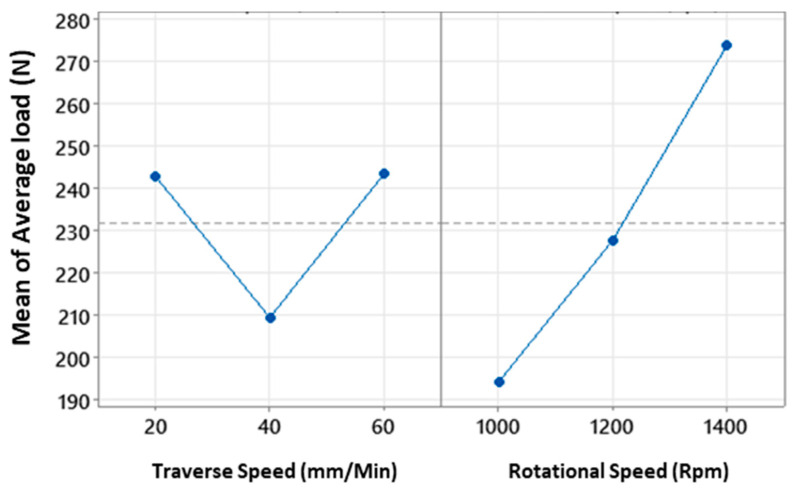
Main effect plots for average load for a confidence level of 95%.

Factorial analysis confirmed that both variables, as well as their interaction, significantly contribute to the average and maximum load. Figure 4 displays the primary contributions of each variable to the average load. Notably, there was a sharp decrease in the average load at a traverse speed of 40 mm/min compared to 20 mm/min. However, as the traverse speed increased from 40 mm/min to 60 mm/min, the average load reverted to a reading similar to that at 20 mm/min. Therefore, within this range, it is optimal to avoid a traverse speed of 40 mm/min, as it notably compromises weld quality. Moreover, a significant improvement in the average load was observed when the rotational speed increased from 1000 rpm to 1400 rpm. The rate of improvement in the average load increased slightly but remained roughly linear. Based on the data analysis, the optimal weld conditions involve a rotational speed of 1400 rpm, paired with a traverse speed of either 20 mm/min or 60 mm/min. For rotational speeds beyond 1400 rpm, there is potential for higher joint strength according to the observed trend, but with diminishing returns based on the affected generated heat and joint structure. Therefore, while increasing rotational speeds beyond 1400 rpm might offer some benefits, the optimal balance between joint strength and heat effects is achieved at 1400 rpm with the specified traverse speeds.

The highest load under these conditions is 336 N, equivalent to 16.54% of the PP strength, with a stress of 3.8 MPa. Shahmiri et al. [16] achieved 20% of the polymer strength when welding AA5052 H34 to PP C30S, with the aluminium on top. Meanwhile, Shiravi et al. [26] reported a joint strength of 2.9 MPa when welding AA5052-H34 to PP-20%talc, also with the aluminium on top. Huang et al. [10] reported a bond strength of 20 MPa after welding the 6061-T6 Al alloy and polyether ether ketone (PEEK). They found 21% of the weaker material’s shear strength when the aluminium alloy was on top [10]

**Figure 5:**
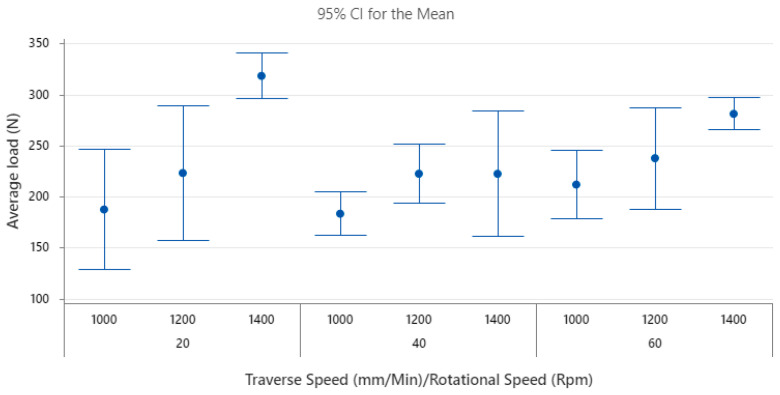
Interval plot of average load (N).

Unlike Huang et al. [10] and Dalwadi et al. [17], the relationship between traverse speed and joint strength in our study is the exact opposite of their results. This discrepancy is mainly due to changes in tool design, which significantly influences the process parameters’ effects. Conversely, a similar relationship between rotational speed and joint strength was observed in the work of Dalwadi et al. [17]. Figure 5 shows that a traverse speed of 60 mm/min typically has the lowest margin of error. This is likely because it is easier to set a high traverse speed on a conventional milling machine, in contrast to lower speeds. This is evident from the high margin of error observed at a 20 mm/min traverse speed. A consistent standard deviation is noted across most parts.

### 3.2. Process Temperatures and Its Relationship with Joint Strength

**Figure 6:**
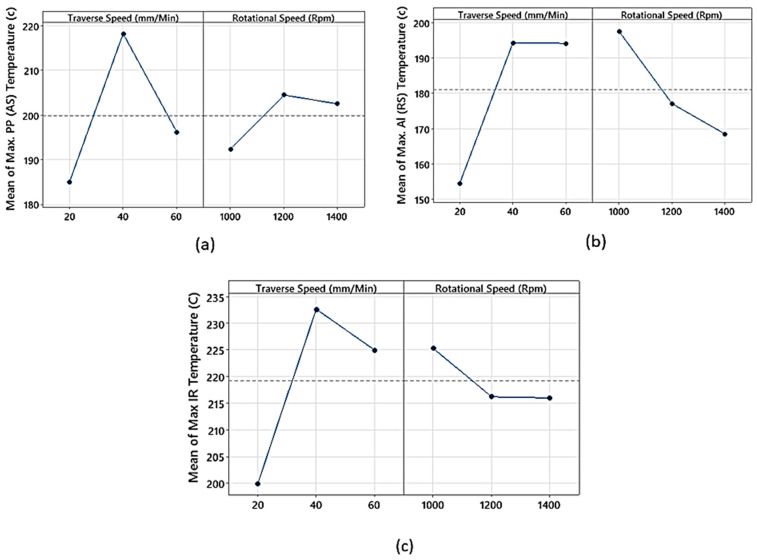
Main effect plots for maximum temperature on: (**a**) the advancing side, (**b**) the retreating side, and (**c**) IR camera temperature reading, for a confidence level of 95%.

In the factorial analysis of temperature, emphasis is placed on the maximum temperature readings acquired from both the advancing and retreating sides, as well as the maximum IR reading. Figure 6a,b depict the primary influence of rotational speed on temperature at the advancing and retreating sides. Although the *p*-value suggests the effect might not be statistically significant, the noted temperature rise is consistent with the established literature. An escalation in rotational speed results in a greater temperature input, causing a gradual increase in temperature. This effect can be ascribed to the reality that an elevated rotational speed amplifies the generation of frictional heat.

On the other hand, when studying the influence of traverse speed, it is evident that the temperature begins to drop after exceeding 40 mm/min. This reduction can be traced back to the diminished contact duration between the tool and the plate, leading to lessened frictional heat generation during swift traversal. Therefore, retaining a moderate traverse speed is advantageous as it ensures ample heat generation through frictional means.

It is also pertinent to mention that the average temperature on the retreating side is notably lower than on the advancing side. This variation can be linked to the greater instantaneous velocity of the tool on the advancing side. This is a result of the compounded effect of the tool’s traverse speed and the tangential speed prompted by rotation; both factors contribute to a heightened relative speed in relation to the sample. The cooler temperature on the retreating side can further be rationalised by accounting for the superior thermal conductivity of aluminium in contrast to polypropylene. Aluminium dissipates heat more efficiently, culminating in cooler temperatures on the retreating side. In summary, while rotational speed influences temperature through augmented frictional heat input, traverse speed modulates temperature by controlling contact duration and heat production.

Figure 6c displays the main effect plot for the maximum surface temperature of the aluminium, as captured by the IR camera. The trends observed for the traverse speed solidify its relationship with the overall temperature. Nevertheless, the rotational speed does not exhibit a significant impact on the temperature, as evidenced by the high *p*-value. To elucidate the link between joint strength and temperature, one can refer to Figure 4. This figure showcases the main effect plot for the load, revealing an inversely proportional relationship with the temperatures shown in Figure 6, while controlling for both the traverse and rotational speeds. The consistency of this correlation across both variables hints that the temperature readings on the advancing side—based on rotational speed—might lack sufficient accuracy. This observation is consistent with findings by Shahmiri et al. [16], who also noted that a surge in heat input corresponded to a decline in joint strength, a result of increased joint defects. To delve deeper into the interaction layer, an SEM examination is utilised, shedding light on the interaction layer’s influence on the comprehensive joint performance.

### 3.3. Joint Morphology

**Figure 7:**
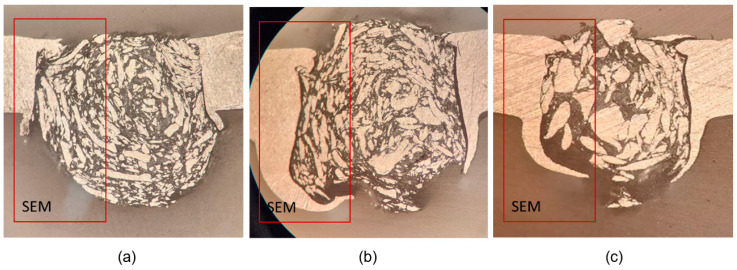
Microscope images of samples: (**a**) #1: 1400 rpm, 20 mm/min, 158 °C (**b**) #2: 1000 rpm, 20 mm/min, 226 °C, and (**c**) #3: 1200 rpm, 60 mm/min, 188 °C.

After the welding process, a specimen from each sample was chosen to study the weld nugget’s structure. The samples labelled #1 (1400 rpm, 20 mm/min), #2 (1000 rpm, 20 mm/min), and #3 (1200 rpm, 60 mm/min) were selected to illustrate the joint morphology, as depicted in Figure 7. These samples showed varied strengths: sample #1 was the strongest, sample #2 was the weakest, and sample #3 demonstrated moderate strength. Different process temperatures and welding parameters influenced the structure of the joint in each sample. The design of the tool featured a flute that produced chips scattered throughout the weld nugget. The size of these chips was directly related to the rotational and traverse speeds. As the rotational speed increased, the cutting tool produced smaller and more precise cuts. In contrast, a higher traverse speed led to larger chips, since the tool had less time for precision, resulting in broader and less accurate cuts. The relationship between traverse speed and rotational speed was directly proportional to the chip size.

In addition to chip distribution, the absence of a cutting edge on the pin’s side caused aluminium hooks to form on both sides of the weld. In these areas, material was cut only in opposition to the tool’s movement. Samples #1, #2, and #3 recorded average temperatures of 158 °C, 226 °C, and 188 °C, respectively. Among them, sample #1 had the lowest temperature, while sample #2 had the highest. Sample #1 also had the lowest v/w ratio at 0.014, whereas sample #3 presented the highest ratio at 0.05. In Figure 7a, sample #1 revealed small and uniformly distributed aluminium chips within the weld nugget. In contrast, Figure 7c displayed larger and more protruding aluminium chips in sample #3, leaving less room for the polypropylene (PP) to integrate. Sample #2 showed a combination of small and elongated, slender chips within its weld nugget.

**Figure 8:**
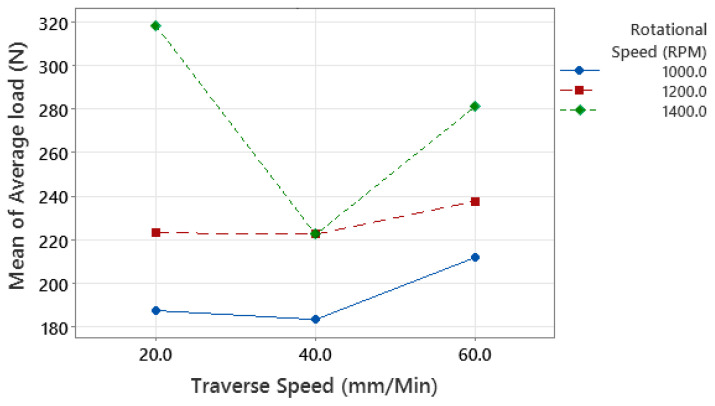
Interaction plot for average load.

The interaction plot for the average load, as shown in Figure 8, offers insights into the relationship between chip size and joint strength. The study reveals that an increase in traverse speed, when considered as a relative value, exerts a more significant influence on chip size. As a result, elevating the chip size through increased traverse speed initially leads to a decrease followed by an increase in joint strength. In contrast, as the rotational speed increases, the strength rises at a more accelerated rate, indicating an inverse relationship with chip size. The hook in sample #1 is straight, distinguishing it from the elliptical shape observed in other samples. This shape of the hook reinforces the joint since the pulling force operates parallel to the overlap. Although the other two samples exhibit similar hook shapes, they differ in thickness.

**Figure 9:**
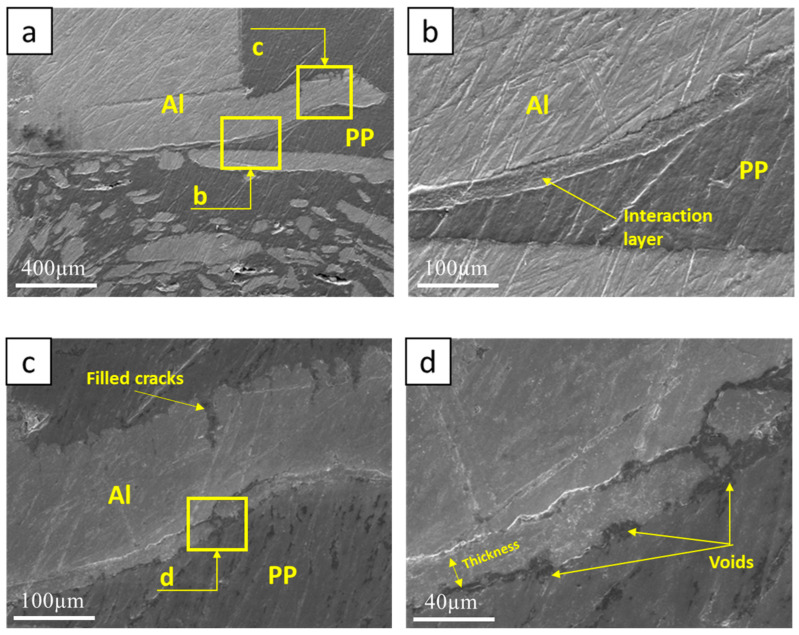
SEM of Sample #1, (**a**) Retreating side Al hook, (**b**) interaction layer on inner RS hook, (**c**) texture on the hook outer side, (**d**) voids at the two sides of the interaction layer.

**Figure 10:**
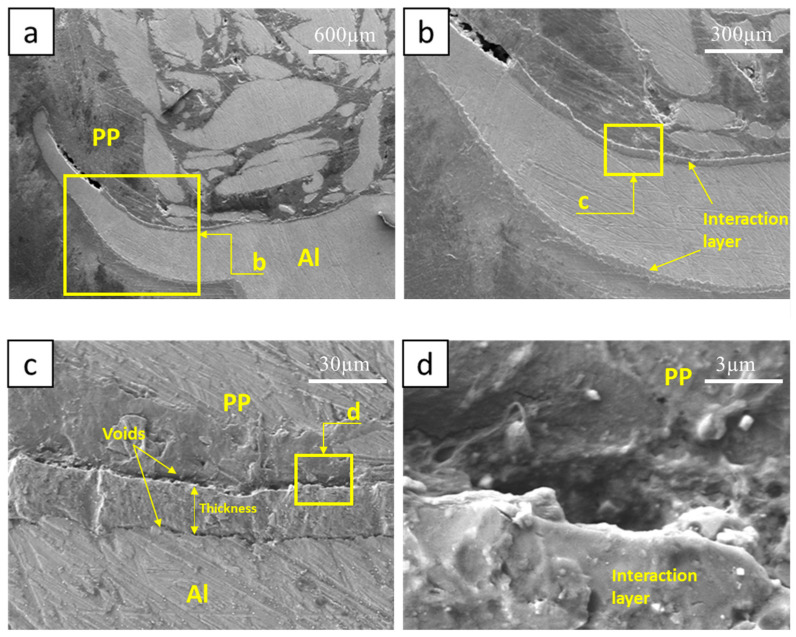
SEM of Sample #3, (**a**) Retreating side Al hook, (**b**) interaction layer on inner and outer sides of RS hook, (**c**) voids at the two sides of the interaction layer, (**d**) voids between the PP and the interaction layer.

**Figure 11:**
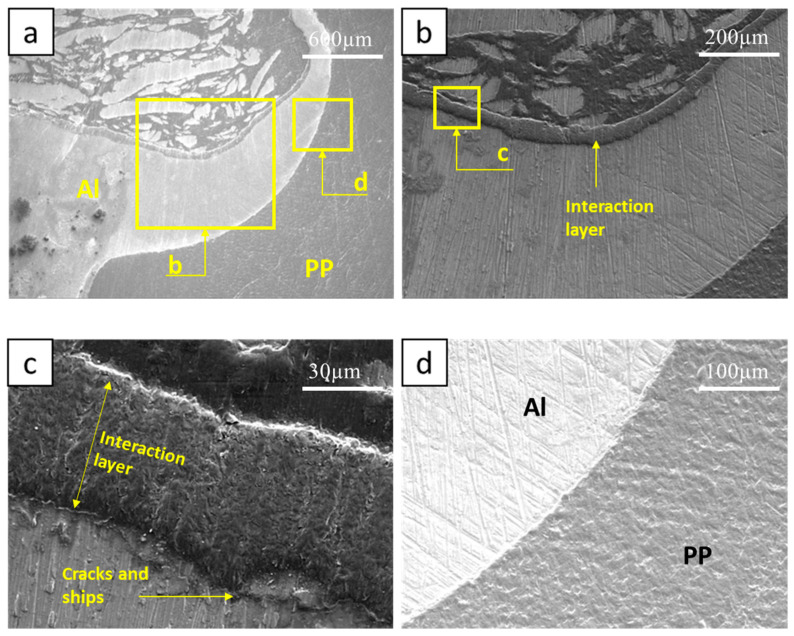
SEM of Sample #2, (**a**) Retreating side Al hook, (**b**) interaction layer on inner side of RS hook, (**c**) zoomed in the interaction layer, (**d**) Al hook outer side.

Figure 9, Figure 10 and Figure 11 display SEM images of samples taken from four distinct positions for each specimen, focusing specifically on the retreating side and the hook, which represents the joint’s fracture point. In Figure 9b, the interaction layer is evident only on the inner side of the hook, whereas cracks appear on the hook’s outer side in Figure 9c. These cracks are filled with PP, enhancing the joint’s strength. The average thickness of the interaction layer, determined from eight separate points, is 26.07 μm. Small voids are noticeable on both sides of the interaction layer, but the cracks within the hook offset their impact.

In contrast to Figure 9 and Figure 10 presents SEM images of Sample 3. Here, the interaction layer is discernible on both sides of the hook, a factor that tends to undermine the joint’s strength. There are no filled cracks visible on the hook, which amplifies the effects of the voids present on both sides of the interaction layer, as depicted in Figure 10c,d. Additionally, a pronounced void is evident at the hook’s tip. The average thickness of this sample’s interaction layer is 27.49 μm. Figure 11 offers SEM images of another specimen, where the interaction layer forms solely on the hook’s inner side. Among the samples, this layer is the thickest, registering at 65.74 μm. This sample exhibits no voids. Instead, minor chips are discerned between the hook and the interaction layer, particularly in Figure 11c. Given the absence of filled cracks, these chips compromise the joint’s strength by facilitating a slipping effect on the hook. The hook’s external surface is entirely smooth, devoid of cracks or voids.

Table 4 compiles the characteristics of the joints, the thicknesses of the interaction layers, and the corresponding temperatures. A pronounced variance in the thickness of the interaction layer is apparent between the most robust and the most fragile specimens. Shahmiri et al. [16] validated that a rise in heat input augments the thickness of the interaction layer. As this thickness expands, the joint’s strength diminishes [16]. This relationship is echoed in the current study: with an uptick in temperature, the thickness of the interaction layer swells, leading to a decline in sample strength.

### 3.4. Process Temperatures and Its Relationship to Material Flow

**Figure 12:**
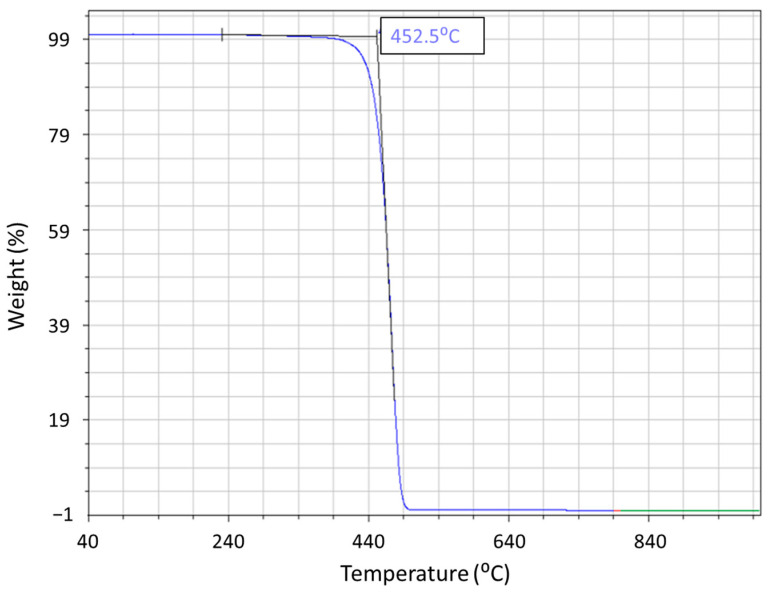
Weight loss vs. temperature for polypropylene.

**Figure 13:**
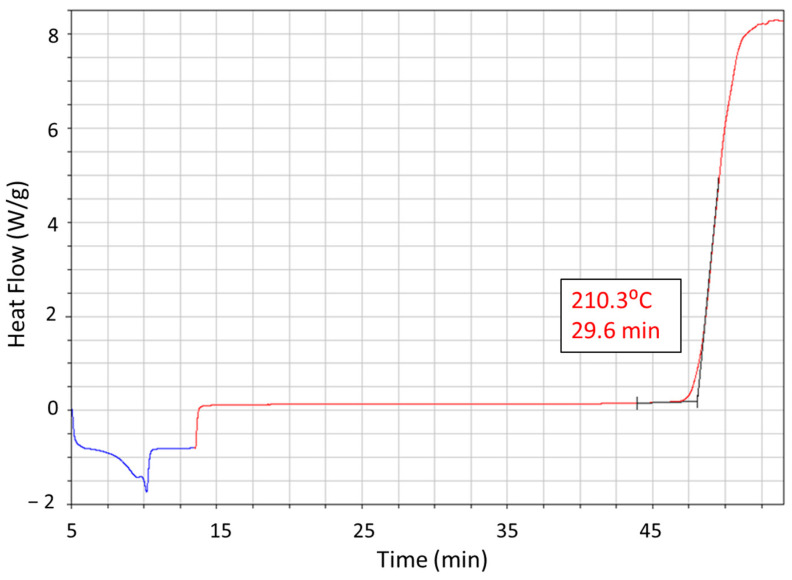
Oxidation induction time of the base polypropylene.

TGA was conducted on the PP base material to examine the point at which the sample undergoes complete degradation. Figure 12 indicates that the weight of the PP material undergoes a one-step breakdown. The one-step breakdown is indicative of a carbon–carbon bond that fosters the random splitting process as the temperature rises [27]. The onset temperature for degradation of the PP material is found to be around 450 °C. This is significantly higher than the maximum FSW process temperature of 250 °C. However, with the combined effects of mechanical shear and temperature, PP degradation (chain scission) may begin at even lower temperatures. The Oxidation Induction Time (OIT) for the PP material at 210 °C is presented in Figure 13. It was measured using Differential Scanning Calorimetry (DSC) and was found to be around 30 min. Commercial PP, on the other hand, has an onset temperature of 235 °C and an induction time of 15 min [28,29]. This suggests that the antioxidant additive package in the PP material used can protect the material from thermo-oxidation for approximately half an hour at the test temperature.

**Figure 14:**
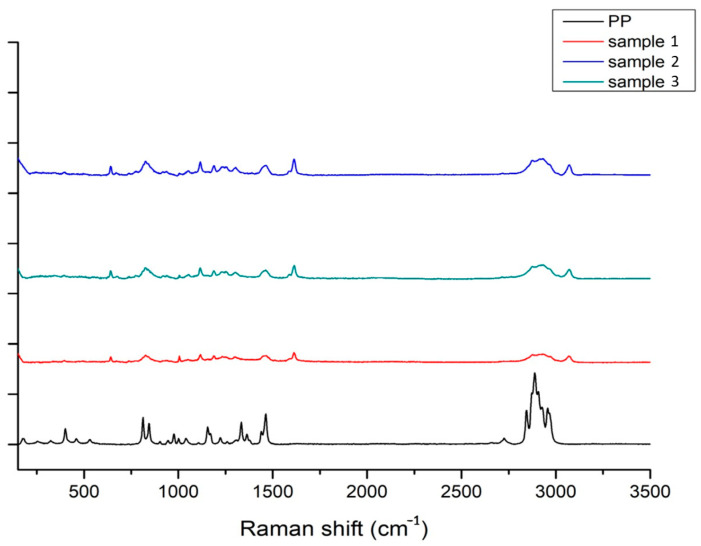
Raman spectrum of the interaction layer of 3 different samples.

Figure 14 displays the Raman spectra of the interaction layer for three selected samples alongside the base PP material. While all samples produced spectra that were largely identical, there were subtle differences in peak intensities. This uniformity suggests a shared chemical structure within the interaction layer of each sample. Raman spectroscopy was employed to evaluate potential sample degradation using characteristic peaks. Table 5 provides insight into the observed Raman spectrum sections. A notable decrease in the peak at 838 $cm^{-1}$, associated with the CH3 group, indicates sample degradation. This reduction can be linked to a diminished number of molecules contributing to this vibrational mode [30]. Using the 842 $cm^{-1}$ peak as a benchmark [31,32], we calculated both the intensity and relative intensity ratios for all samples. The relative intensity ratio spanned between 20 and 30 for all samples. An uptick in this ratio typically points to polymer degradation, a process in which polymer chains break down at higher temperatures and under mechanical stress, leading to the formation of degradation by-products such as aromatics, carbonyl groups, and hydroxyl groups. Yet, the absence of peaks between 1600–1800 $cm^{-1}$, typical of the C=O stretching mode, with an exception for a peak at 1618 $cm^{-1}$ related to C=C stretching [33,34], suggests that polypropylene degradation took place without a thermo-oxidation process, and as a result, no carbonyl groups were formed. This phenomenon is likely due to the antioxidants in the polypropylene’s additives, which inhibit oxidative reactions. The TGA analyses corroborate this, showing a degradation onset temperature of 450 °C, which is significantly above the maximum temperature observed during FSW. Additionally, the Oxidation Induction Time (OIT) analyses via DSC show that the consumption of antioxidants and the thermo-oxidative degradation of polypropylene only commence after 30 continuous minutes of exposure to 100% O_2_ at 210 °C. Considering the FSW’s processing time under atmospheric conditions (21% O_2_), thermo-oxidative degradation seems improbable. Han et al. [35] utilised XPS to confirm the creation of C-O-Al bonds, enhancing the bond between the aluminium (Al) and polypropylene (PP) interface. These bonds are a product of reactions between C=O groups and the oxides present on the Al surface [35,36]. The lack of the C=O stretching mode suggests that this particular reaction was absent, negating the likelihood of intermetallic compound formation.

In investigating the role of mechanical interlocking as the dominant factor in joint strength, Electron Dispersive Spectroscopy (EDS) mapping was executed at the aluminium and PP interface. This technique was deployed to identify the presence of three pivotal elements—carbon, oxygen, and aluminium—at the specified point of interest, providing insights into the nature of the bond. A distinct peak at 1617 $cm^{-1}$ observed in the interaction layer has been previously attributed to the C=C bond. However, alternate hypotheses suggest its association with C=O or Al-Mg-Si bonds. Using EDS, we aimed to clarify this association [37,38,39].

**Figure 15:**
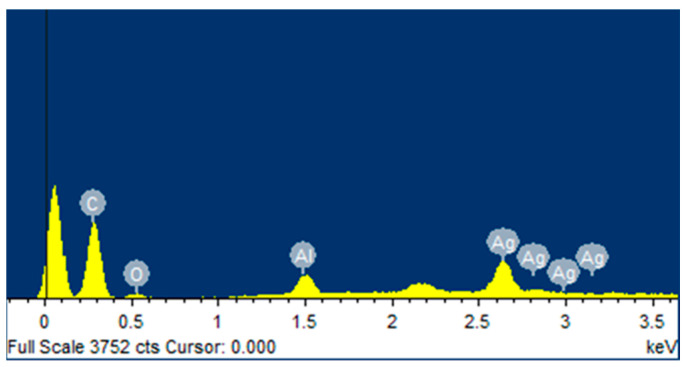
EDS spectrum of the interaction layer (Sample #1).

**Figure 16:**
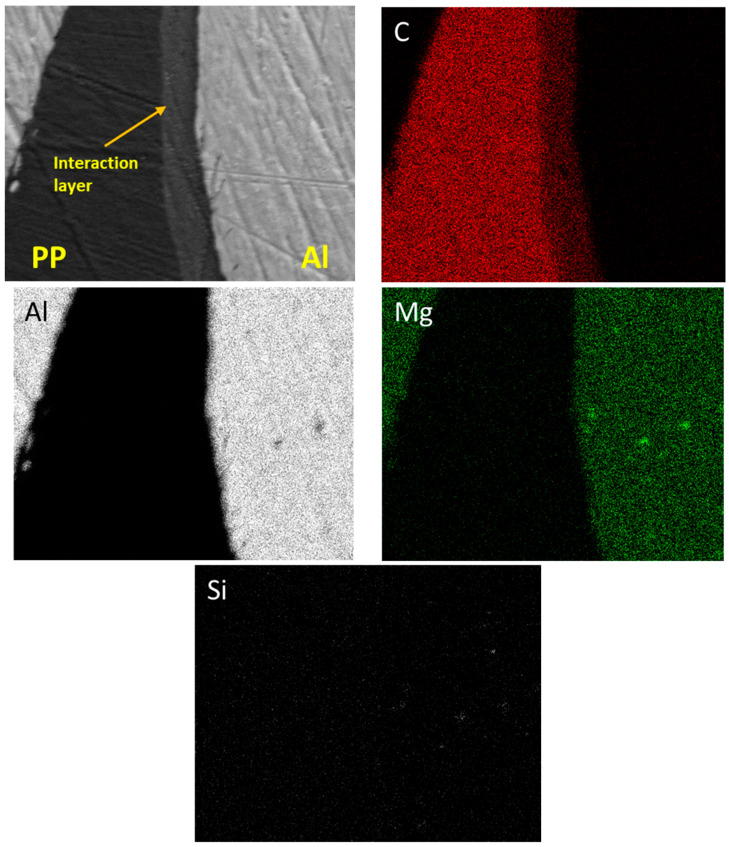
EDS elemental mapping of Al-PP interface.

Figure 15 presents a spectrum from the interaction layer of sample #1, verifying the presence of carbon, oxygen, and aluminium. It is noteworthy that the oxygen concentration is markedly reduced, and silicon is conspicuously absent. EDS mapping in Figure 16 indicates silicon’s presence primarily as background noise, lending further credence to the assertion that the peak at 1617 $cm^{-1}$ is closely related to C=C, rather than Al-Mg-Si or C=O. The conspicuous absence of mixed aluminium compounds within the interaction layer further negates the possibility of the presence of a C-O-Al bond.

In conclusion, all the samples exhibited a similar chemical structure, suggesting that the chemical composition does not have a significant influence on joint strength. The differences in joint strength are likely due to mechanical interlocking, with the chemical composition playing a secondary role. Although the structure of the interaction layer was consistent across all samples, variations in thickness were observed. This observation is corroborated by SEM analysis and is consistent with existing literature that associates increased thickness with reduced joint strength [16].

**Table 5:** Raman spectrum ranges and their description.

$\mathrm{Raman}\;\mathrm{Shift}\;(cm^{-1}$)	Description
200–800	Most of the peaks are related to the alloy. The changes offer information about the bonding, interaction, and oxide formation adhesion on the alloy [40]
800–900	Changes offer insights into the thermal stability of polypropylene and its degree of degradation [41]
900–1000	C-C stretching mode which is sensitive to the degree of crystallinity in the polymer [31]
1300–1550	Mainly corresponds to C-H deformation vibrations [32].
1300–1460	$CH_{2}$ deformation modes and $CH_{3}$ bending modes
1460–1550	Aromatic ring stretching mode in the polypropylene molecule.
2850–3000	The $CH_{2}$ symmetric and asymmetric stretching modes: sensitive to the degree of crystallinity in the polymer.

## 4. Conclusions

The feasibility of welding a novel hybrid structure of AA5052 and Polypropylene (PP) with the metal on top was thoroughly examined. To validate and justify the results, ANOVA, chemical analysis, and microstructure analysis were performed. The study was conducted keeping certain parameters and tool designs constant, including the new tool design, plunge depth, tilt angle, and welding configuration. In contrast, the rotational speed and traverse speed were altered to determine the optimal joining condition using the new tool design.

1. Efficacy of Novel Tool Design: The novel tapered fluted pin tool design demonstrated its effectiveness in this study. Its unique configuration enabled successful penetration of the AA5052 alloy, establishing a solid foundation for the welding process and ensuring a commendable weld initiation.
2. Optimal Process Parameters: The most favourable conditions for joining AA5052 and PP, using the novel tapered fluted pin, are realised with a rotational speed of 1400 RPM and a traverse speed of 20 mm/min. Under these conditions, the joint exhibited a load of 336 N and achieved a tensile strength of 3.8 MPa. In relative terms, this strength corresponds to 16.54% of the weak material’s inherent strength. To put this in perspective, other studies noted joint strengths of 2.9 MPa for AA5052-H34 to PP-20%talc [26], and a relative strength of 21% when fusing 6061-T6 Al alloy with polyether ether ketone (PEEK) [10].
3. Parameter Influence on Tensile Strength: The tensile strength of the joint is directly modulated by both rotational and traverse speeds. Notably, an increase in rotational speed positively impacts joint strength, whereas traverse speed shows a non-linear influence with an initial decrement at 40 mm/min, followed by an enhancement.
4. Temperature Dynamics: The study elucidated that traverse speed affects the weld’s temperature, exhibiting a decrease after a certain threshold is reached. The rotational speed, on the other hand, demonstrated minimal influence on this parameter.
5. Microstructure Insights: Microstructural analysis of the welded region revealed a composite material composed of aluminium chips and PP, reinforced by the presence of two distinct aluminium hooks. The fractures were predominantly observed to initiate at the interface of the retreating side hook.
6. Predominance of Mechanical Interlocking: Through advanced characterisation, it was discerned that mechanical interlocking is the preeminent contributor to joint strength variations. The absence of peaks between 1600–1800 $cm^{-1}$ in the Raman spectrum, typically indicative of C=O stretching mode, suggests that PP degradation transpired without a thermo-oxidation process. This is further supported by corroborative EDS mapping data, which negate the existence of C-O-Al bonds. The presence of antioxidants in PP’s additives probably inhibited oxidative reactions.
7. Joint Strength Dependencies: Joint strength exhibits an inverse dependency on heat input, predominantly resulting from fluctuations in the interaction layer’s thickness.
8. Interaction Layer Dynamics: Consistency in the chemical structure was observed across samples, suggesting that the chemical composition was not a major factor influencing joint strength. However, the thickness of the interaction layer emerged as a crucial determinant of joint strength. A direct inverse relationship was observed between increasing thickness, attributed to increased heat input, and decreasing joint strength. Moreover, PP-filled cracks within the hook were found to significantly enhance joint strength, as confirmed by SEM analysis.

In essence, this comprehensive study highlights the importance of mechanical interlocking in determining joint strength. It also sheds light on the potential for further enhancements in welding hybrid structures of polymer–metal, such as AA5052 and PP, using a fluted pin design.

## Figures and Tables

**Table 1:** Physical properties of AA5052 and polypropylene.

Base Material	AA 5052	Polypropylene
Density (kg/m^3^)	2680	905
Thermal conductivity (W/m·K)	138	0.24
Ultimate tensile strength (MPa)	210–260	>25
Shear strength (MPa)	180	25

**Table 2:** Chemical composition of AA 5052.

Chemical Composition of AA 5052 (%)								
Si	Fe	Cu	Mn	Mg	Cr	Zn	Ti	Aluminium (Minimum)
0.12	0.31	0.003	0.037	2.5	0.172	0.001	0.016	96.841

**Table 3:** Full factorial design of the experiment.

Fixed Parameters	Value		
Pin Shape	Fluted 4 mm pin (straightened self-tapping screw)		
Pin length (mm)	4		
Tool Shoulder (mm)	15		
Plunge Depth (mm)	0.1		
Tilt Angle (Degree)	2		
**Variables**	**Level 1**	**Level 2**	**Level 3**
Rotation Speed (RPM)(CW)	1000	1200	1400
Traverse speed (mm/min)	20	40	60

**Table 4:** Joints features and interaction layers thickness.

Sample #	w (RPM)	v (mm/min)	Joint Features	Interaction Layer Thickness (μm)	Joint Temperature (°C)
1 (Strongest)	1400	20	Hook’s PP filled cracks, voids, small Al chips in the weld nugget	26.07	158
2 (Weakest)	1000	20	Chipped hook inner side, thick interaction layer, slim and long Al chips in the weld nugget	65.74	226
3	1200	60	Voids, two-sided interaction layer, bulk Al chips in the weld nugget	27.49	188

## Data Availability

Data is contained within the article.

## References

[1] Ghori S.W., Rasheed M., Saba N., Jawaid M. (2018). The role of advanced polymer materials in aerospace. Sustainable Composites for Aerospace Applications.

[2] Ramarathnam G., Libertucci M., Sadowski M.M., North T.H. (1992). Joining of polymers to metal. Weld. J. N. Y..

[3] Zheng K., Politis D.J., Wang L., Lin J. (2018). A review on forming techniques for manufacturing lightweight complex—Shaped aluminium panel components. Int. J. Light. Mater. Manuf..

[4] Haghshenas M., Khodabakhshi F. (2019). Dissimilar friction-stir welding of aluminum and polymer: A review. Int. J. Adv. Manuf. Technol..

[5] Khodabakhshi F., Haghshenas M., Chen J., Amirkhiz B.S., Li J., Gerlich A. (2016). Bonding mechanism and interface characterisation during dissimilar friction stir welding of an aluminium/polymer bi-material joint. Sci. Technol. Weld. Join..

[6] Shankar S., Kaushal A., Chattopadhyaya S., Vilaça P., Bennis F. (2021). Joining of aluminium to polymer by friction stir welding: An overview. IOP Conf. Ser. Mater. Sci. Eng..

[7] Moshwan R., Rahmat S.M., Yusof F., Hassan M.A., Hamdi M., Fadzil M. (2015). Dissimilar friction stir welding between polycarbonate and AA 7075 aluminum alloy. Int. J. Mater. Res..

[8] Ghiya R., Badheka V.J. (2021). A review of friction stir lap welding of polymer to metal. Polym. Technol. Mater..

[9] Ullegaddi K., Murthy V., Harsha R.N., Manjunatha (2017). Friction Stir Welding Tool Design and Their Effect on Welding of AA-6082 T6. Mater. Today Proc..

[10] Huang Y., Meng X., Wang Y., Xie Y., Zhou L. (2018). Joining of aluminum alloy and polymer via friction stir lap welding. J. Am. Acad. Dermatol..

[11] Mahakur V.K., Gouda K., Patowari P.K., Bhowmik S. (2021). A Review on Advancement in Friction Stir Welding Considering the Tool and Material Parameters. Arab. J. Sci. Eng..

[12] Ratanathavorn W., Melander A. (2015). Dissimilar joining between aluminium alloy (AA 6111) and thermoplastics using friction stir welding. Sci. Technol. Weld. Join..

[13] Derazkola H.A., Elyasi M. (2018). The influence of process parameters in friction stir welding of Al-Mg alloy and polycarbonate. J. Manuf. Process..

[14] Rai R., De A., Bhadeshia H., DebRoy T. (2011). Friction stir welding tools. Sci. Technol. Weld. Join..

[15] Trimble D., O’Donnell G.E., Monaghan J. (2015). Characterisation of tool shape and rotational speed for increased speed during friction stir welding of AA2024-T3. J. Manuf. Process..

[16] Shahmiri H., Movahedi M., Kokabi A.H. (2016). Friction stir lap joining of aluminium alloy to polypropylene sheets. Sci. Technol. Weld. Join..

[17] Dalwadi C.G., Patel A.R., Kapopara J.M., Kotadiya D.J., Patel N.D., Rana H. (2018). Examination of mechanical properties for dissimilar friction stir welded joint of Al alloy (AA-6061) to PMMA (Acrylic). Mater. Today Proc..

[18] Derazkola H.A., Khodabakhshi F., Simchi A. (2017). Friction-stir lap-joining of aluminium-magnesium/poly-methyl-methacrylate hybrid structures: Thermo-mechanical modelling and experimental feasibility study. Sci. Technol. Weld. Join..

[19] Zhang Y.N., Cao X., Larose S., Wanjara P. (2012). Review of tools for friction stir welding and processing. Can. Met. Q..

[20] Panneerselvam K., Lenin K. (2012). Investigation on effect of tool forces and joint defects during FSW of polypropylene plate. Procedia Eng..

[21] Sandeep R., Arivazhagan N. (2021). Innovation of thermoplastic polymers and metals hybrid structure using friction stir welding technique: Challenges and future perspectives. J. Braz. Soc. Mech. Sci. Eng..

[22] Upadhyay P., Hovanski Y., Fifield L.S., Simmons K.L. (2015). Friction stir lap welding of aluminum—Polymer using scribe technology. Friction Stir Welding and Processing VIII.

[23] (2019). Standard Test Method for Apparent Shear Strength of Single-Lap-Joint Adhesively Bonded Metal Specimens by Tension Loading (Metal-to-Metal).

[24] (2022). Plastics—Thermogravimetry (TG) Of Polymers—Part 1: General Principles.

[25] (2018). Plastics—Differential Scanning Calorimetry (DSC)—Part 6: Determination of Oxidation Induction Time (Isothermal OIT) and Oxidation Induction Temperature (Dynamic OIT).

[26] Shiravi H., Movahedi M., Ozlati A. (2022). Improving appearance and mechanical strength of aluminum-polypropylene/talc composite friction stir joint using a novel tool design. Int. J. Adv. Manuf. Technol..

[27] Miandad R., Rehan M., Barakat M.A., Aburiazaiza A.S., Khan H., Ismail I.M.I., Dhavamani J., Gardy J., Hassanpour A., Nizami A.-S. (2019). Catalytic pyrolysis of plastic waste: Moving toward pyrolysis based biorefineries. Front. Energy Res..

[28] Zhang G., Nam C., Chung T.C.M. (2017). Developing polypropylene bonded hindered phenol antioxidants for expanding polypropylene applications in high temperature conditions. J. Mater. Sci. Eng..

[29] Zhang G., Nam C., Petersson L., Jämbeck J., Hillborg H., Chung T.C.M. (2018). Increasing Polypropylene High Temperature Stability by Blending Polypropylene-Bonded Hindered Phenol Antioxidant. Macromolecules.

[30] Guo X., Lin Z., Wang Y., He Z., Wang M., Jin G. (2019). In-Line Monitoring the Degradation of Polypropylene under Multiple Extrusions Based on Raman Spectroscopy. Polymers.

[31] de Báez M.A., Hendra P., Judkins M. (1995). The Raman spectra of oriented isotactic polypropylene. Spectrochim. Acta Part A Mol. Biomol. Spectrosc..

[32] Gopanna A., Mandapati R.N., Thomas S.P., Rajan K., Chavali M. (2018). Fourier transform infrared spectroscopy (FTIR), Raman spectroscopy and wide-angle X-ray scattering (WAXS) of polypropylene (PP)/cyclic olefin copolymer (COC) blends for qualitative and quantitative analysis. Polym. Bull..

[33] Lin-Vien D., Colthup N.B., Fateley W.G., Grasselli J.G. (1991). Compounds Containing the Carbonyl Group. The Handbook of Infrared and Raman Characteristic Frequencies of Organic Molecules.

[34] Blakey I., George G.A. (2000). Raman spectral mapping of photo-oxidised polypropylene. Polym. Degrad. Stab..

[35] Han S., Wu L.H., Jiang C.Y., Li N., Jia C.L., Xue P., Zhang H., Zhao H.B., Ni D.R., Xiao B.L. (2020). Achieving a strong polypropylene/aluminum alloy friction spot joint via a surface laser processing pretreatment. J. Mater. Sci. Technol..

[36] Wang W., Wang S., Zhang X., Xu Y., Tian Y., Huang H. (2021). Enhanced aluminum alloy-polymer friction stir welding joints by introducing micro-textures. Mater. Lett..

[37] Wang S., Xu Y., Wang W., Tian Y., Zhang X., Huang H., Zheng D. (2022). Enhancing interfacial bonding in friction stir lap welding of light metal and carbon fiber reinforced polymer composite. J. Manuf. Process..

[38] Liu F., Dong P., Pei X. (2020). A high-speed metal-to-polymer direct joining technique and underlying bonding mechanisms. J. Am. Acad. Dermatol..

[39] Liu F., Dong P., Lu W., Sun K. (2019). On formation of Al O C bonds at aluminum/polyamide joint interface. Appl. Surf. Sci..

[40] Adar F. (2014). Raman spectra of metal oxides. Spectroscopy.

[41] Andreassen E. (1999). Infrared and Raman spectroscopy of polypropylene. Polypropylene.

